# Modeling Integrated Cellular Machinery Using Hybrid Petri-Boolean Networks

**DOI:** 10.1371/journal.pcbi.1003306

**Published:** 2013-11-07

**Authors:** Natalie Berestovsky, Wanding Zhou, Deepak Nagrath, Luay Nakhleh

**Affiliations:** 1 Department of Computer Science, Rice University, Houston, Texas, United States of America; 2 Department of Bioengineering, Rice University, Houston, Texas, United States of America; 3 Department of Chemical and Biomolecular Engineering, Rice University, Houston, Texas, United States of America; Princeton University, United States of America

## Abstract

The behavior and phenotypic changes of cells are governed by a cellular circuitry that represents a set of biochemical reactions. Based on biological functions, this circuitry is divided into three types of networks, each encoding for a major biological process: signal transduction, transcription regulation, and metabolism. This division has generally enabled taming computational complexity dealing with the entire system, allowed for using modeling techniques that are specific to each of the components, and achieved separation of the different time scales at which reactions in each of the three networks occur. Nonetheless, with this division comes loss of information and power needed to elucidate certain cellular phenomena. Within the cell, these three types of networks work in tandem, and each produces signals and/or substances that are used by the others to process information and operate normally. Therefore, computational techniques for modeling integrated cellular machinery are needed. In this work, we propose an integrated hybrid model (IHM) that combines Petri nets and Boolean networks to model integrated cellular networks. Coupled with a stochastic simulation mechanism, the model simulates the dynamics of the integrated network, and can be perturbed to generate testable hypotheses. Our model is qualitative and is mostly built upon knowledge from the literature and requires fine-tuning of very few parameters. We validated our model on two systems: the transcriptional regulation of glucose metabolism in human cells, and cellular osmoregulation in *S. cerevisiae*. The model produced results that are in very good agreement with experimental data, and produces valid hypotheses. The abstract nature of our model and the ease of its construction makes it a very good candidate for modeling integrated networks from qualitative data. The results it produces can guide the practitioner to zoom into components and interconnections and investigate them using such more detailed mathematical models.

## Author Summary

Within the cell of an organism, three networks—signaling, transcriptional, and metabolic—are always at work to determine the response of the cell to signals from its environment, and consequently, its fate. Evidence from experimental studies is painting a picture of complex crosstalk among these networks. Thus, while a wide array of computational techniques exist for analyzing each of these network types, there is clear need for new modeling techniques that allow for simultaneously analyzing integrated networks, which combine elements from all three networks. Here, we provide a step towards achieving this task by combining two population modeling techniques—Petri nets and Boolean networks—to produce an integrated hybrid model. We demonstrate the accuracy and utility of this model on two biological systems: transcriptional regulation of glucose metabolism in human cells, and cellular osmoregulation in yeast.

## Introduction

While the genome contains all hereditary information, the decisions that a cell makes are governed by a complex cellular machinery that resides above the genome. Modeling this machinery is both important—as it helps understand proper cellular functioning and the implications of aberrations thereof, and a daunting—given the “known unknowns” (e.g., kinetic parameters of given reactions) and the “unknown unknowns” (data incompleteness is the rule, rather than the exception, in biological research).

The cellular machinery can be broken down into three main components—signaling, transcription regulation, and metabolism—each of which consists of a network of molecules and interactions among them. The signaling network is responsible for relaying messages from the external environment of a cell to the nucleus. Inside the nucleus, the transcription regulation network determines, upon receiving signals, which genes are expressed, and to what extent. The metabolic network is the energy and resource management component of the cell, producing energy and products that are required by cellular processes. Various modeling techniques have been used successfully for modeling the dynamics of each of these components individually.

The success of modeling each of the three components individually notwithstanding, these components are interconnected within the cell and their dynamics are intertwined, thus creating a complex network whose modeling and understanding are major endeavors in systems biology. Several biological studies and surveys have highlighted this interconnection inside the cell and the significance of analyzing the components simultaneously rather than individually, including, but not limited to, [1]–[6].

Indeed, several approaches were introduced recently for integrated modeling of biological networks: regulatory FBA (rFBA) [7], steady state regulatory FBA (SR-FBA) [8], integrated FBA (iFBA) [9], integrated dynamic FBA (idFBA) [10], probabilistic regulation of metabolism (PROM) [11], the method of [12], dynamic FBA (dFBA) [13] and a recently published whole cell computational model [14]. One common aspect to all the existing models is the use of flux balance analysis (FBA) for modeling carbon and energy metabolism. FBA is a widely used method that estimates fluxes of metabolic reactions, thereby making it possible to predict the growth rate of an organism or the rate of production of a metabolite of interest. However, FBA is only suitable for determining fluxes at steady state. With exceptions of some modified forms, FBA does not account for regulatory effects such as activation of enzymes by protein kinases or regulation of gene expression [15]. The methods that use the unmodified version of FBA – all but idFBA and dFBA — only capture the steady state of metabolism, therefore not capturing the full dynamic within the cell. These methods mainly acquire the effects of changes that individual components have on each other. On the other hand, the methods that discretize FBA (dFBA and idFBA), are able to reveal not only a more complete profile of the cell, but also the dynamic behavior of the interconnections between the components. For recent surveys of these methods, please see [16], [17].

In this paper, we propose a new Integrated Hybrid Model (IHM) that aims to capture the dynamic behavior within and between the components of the cell, and which belongs to the class of executable models [18]. This model integrates two types of modeling techniques: *Petri nets* (PNs), which have been used for modeling metabolic networks and signaling networks [19], and *Boolean networks*, which have been used to model regulatory networks as well as protein signaling networks [20], [21]. One of the first successful Petri net-based models of metabolism was devised by Reddy *et al.* [22], [23]. Over the recent years, various types of Petri nets have been introduced and extensively used in modeling different metabolic systems [24]–[27]. Signaling pathways, on the other hand, have posed more of a challenge for Petri nets. Their highly interleaved (with possible forward- and backward loops) and parametrized nature makes it a difficult mapping onto a Petri net framework. Despite these limitations, Petri nets have been shown to be applicable in signaling pathways using careful parameterization and execution strategies [28]–[32]. Transcription regulation has been modeled successfully using Boolean networks, starting with the work of [33]. Over the years, with the steady increase in the amount of data on genetic regulation, Boolean networks became a common strategy for modeling this cellular process; e.g., [34]–[36].

Our integrated hybrid model uses Petri nets to model the metabolic and signaling components, and Boolean networks to model the transcriptional component. Further, the model makes connections between the Petri net and Boolean network component using a special modeling part. Our modeling approach assumes knowledge of the connectivity among the various species in the system, and is then minimally parameterized based on qualitative data. The dynamics of the biological system are then obtained by executing the parametrized model. Of the existing approaches, idFBA is comparable to our approach, as it allows for modeling the dynamics by discretizing time and conducting FBA analyses for short time intervals. However, idFBA is applicable where FBA models have been curated (e.g., for single-cell organisms), whereas our modeling approach is applicable more broadly in terms of organism selection, and requires only qualitative data.

We implemented and tested our modeling methodology on two biological systems: (1) the transcriptional regulation of glucose in human physiology, with knowledge based on [1], and (2) osmoregulation in *S. cerevisiae*, based on the system in [37]. The two systems differ in temporal and spatial scales. For the transcriptional regulation of glucose, the interactions among different components are reflected in the cooperation among multiple cell types, and the mass transportation is through blood vessels in the human body, thus acting at longer time scales than single cell systems. On the other hand, the modeling of osmoregulation in *S. cerevisiae* encompasses metabolism, signaling and transcriptional regulation, all within a single cell. The exchange of proteins or metabolites is mediated through diffusion and cellular transportation. We choose the two systems to show the diversity of the biological scenarios to which our integrated hybrid model is applicable. The two systems are very well curated and studied, both experimentally and computationally. This makes them ideal for validating our methodology and for comparing with existing modeling frameworks. Our modeling approach produced results that match experimentally derived data (in terms of both validation and prediction). There is an abundance of qualitative data on biological interaction networks, and developing models and methods that utilize such data is desirable. Our proposed method fits within this category which offers a complementary approach, rather than an alternative one, to the FBA-based category of methods as well as other categories such as kinetics-based methods.

## Methods

Our integrated hybrid model combines two modeling techniques, Petri nets and Boolean networks. We begin by briefly reviewing each of these models, and their use in modeling biological networks, and then describe the new integrated hybrid model.

### Petri nets and their execution

**Figure 1:**
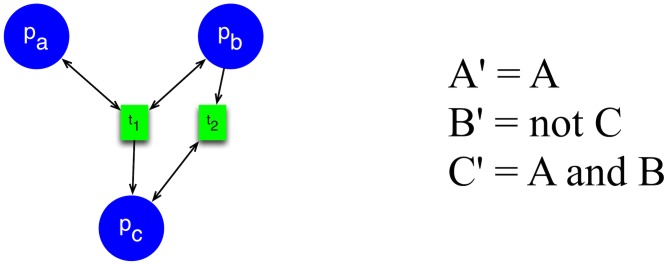
Illustration of Petri nets and Boolean networks. Consider a cellular network that involves three molecular species 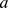, 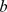 and 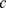, where 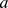 is self-regulatory (activating), 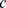 inhibits 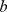, and both 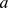 and 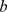 activate 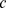 in a cooperative manner. (Left) A Petri net representation, with three places corresponding to the molecular species, and two transitions corresponding to the reactions. A *read arc* (line with arrows on both ends) connecting place 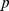 to transition 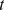 means that when transition 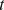 fires, the number of tokens in place 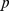 does not change. Notice that the inhibition of 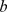 is represented by transition 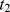 which consumes tokens from 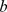. (Right) A Boolean network representation, with three Boolean variables corresponding to the molecular species. The primed version of a variable indicated the next-state of that variable. In other words, these Boolean formulas can be interpreted a 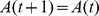, 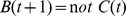, and 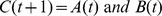.

In our context, a Petri net (PN) is a 4-tuple 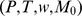 that defines a weighted, complete, directed, bipartite graph. The disjoint sets 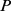 and 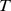 correspond to two types of nodes, *places* and *transitions*, respectively. In modeling signal transduction and metabolism, they correspond to chemical species and biochemical reactions that happen among these species. The element 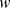 is a mapping defined 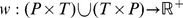, where 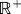 is the set of non-negative real numbers. These mappings could be used to encode, for example, stoichiometries of biochemical reactions. Finally, 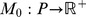 is the initial marking of the Petri net, which assigns a number of *tokens* to each place. This correspond to the initial concentration of chemical species. The state of a Petri net is given by a vector 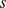 of length 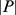 with 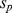 being the number of tokens in place 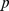. In particular, the *initial state*, 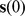, is given by the initial marking 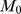. Additionally, a vector 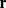 of length 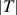 provides the transition rates for the system, where 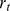 denotes the rate of transition 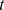 to simulate the empirical rate constant used in the law of mass action that governs the corresponding reaction. The Petri net can be executed both deterministically and stochastically [38]–[40]. In this work, we utilize a stochastic protocol based on the Gillespie “first reaction” method [41]. The method characterizes the dynamics of each transition 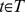 by a *propensity function* 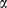. Let 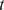 be a transition whose inputs is the set 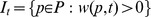 and outputs is the set 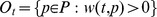. In state 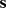, the propensity 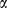 of transition 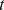 is defined by
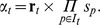
Given these propensity values, the method determines the putative time 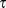 at which the next transition fires based on the probability distribution function given by
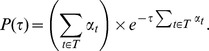
The transition with the smallest time 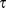 is then chosen to fire. Firing transition 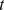 amounts to updating the number of tokens in every place 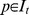 according to the rule 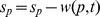 and updating the number of tokens in every place 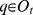 according to the rule 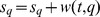. Once a transition is executed, the state of the Petri net changes. The execution time is updated by 1, which is, in our case, a slight modification from the original algorithms where time is updated by 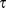. Consecutive firings of transitions results in a walk through the state space of the Petri net from the start state 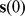. The final dynamics of the system is acquired by averaging several full runs of Gillespie starting from the initial state 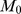 and executing the same number of steps. A detailed description of Petri nets and its application to systems biology can be found in [19]. See Figure 1 for an illustration.

### Boolean networks and their execution

A Boolean network is a 3-tuple 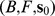, where 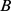 is a vector of 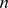 Boolean variables (that is, variables that take values in the set 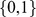) and 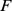 is a vector of 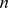 Boolean functions with function 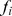, for 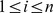, associated with variable 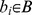, and 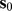 is a vector of length 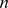 that has a Boolean value for each of the 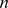 variables and denotes the start state. In modeling transcriptional regulation, each Boolean variable indicates whether a gene is being transcribed at a given time and the Boolean functions stipulate how transcriptional factors regulate the transcription of their targets. The state of a Boolean network is a Boolean vector 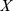 of size 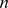, where 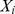 is the value of variable 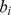. The value of 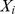 of variable 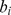 is updated by applying function 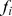 to the current state of the Boolean network. More formally, let 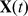 be the state of the Boolean network at time 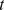. Then, if function 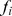 is executed at time 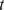, the state of the Boolean network one step later is given by 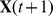, where 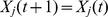 for every 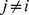, and 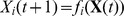. In particular, 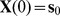. Given a Boolean network representing a set of variables, the dynamics of the system can be simulated by repeatedly executing the Boolean functions and updating the “current” state. In the classical synchronous simulation, the states of all variables are updated simultaneously *after all* of the functions in 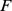 have executed. In an asynchronous simulation, only one Boolean function is chosen and executed in a given time step. See Figure 1 for an illustration.

### The integrated hybrid model and its execution

As described above, gene regulatory networks have been successfully modeled using Boolean networks. Signaling and metabolic networks have been successfully modeled using Petri nets. In our integrated hybrid model, the regulatory components of the biological system are modeled using Boolean networks, whereas the other two components are modeled using Petri nets. To facilitate connections between the two components, our model contains, in addition to the Petri net and Boolean network components, a set of Place-to-Boolean and Boolean-to-Place triplets that create a Boolean value based on binarization of the number of tokens and a number of tokens based on a Boolean value, respectively. We now describe our modeling approach formally.

#### Syntax

The integrated hybrid model (IHM) is a 4-tuple tuple 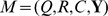 where:

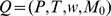
 is a Petri net.

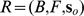
 is a Boolean network where each Boolean function 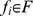 takes as input the state of variables in 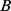.

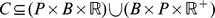
 is a set of triplets that connect places in the Petri net component with Boolean variables in the Boolean network component.

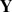
 is an initial marking of 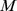 such that that
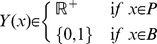

It is important to note that the two sets of variables, 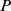 and 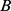, are disjoint. In our approach, we model the metabolic and signaling components using a single Petri net, and the transcriptional regulation component using a single Boolean network. The set of triplets in 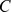 is defined for the places and Boolean variables that provide the connections at the interface of the biological components. The choice of these triplets vary from one biological system to another.

#### Semantics

Given an IHM 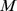, it is now straightforward to execute it and produce the dynamics of the system, as each of the two components of the model is amenable to both deterministic and stochastic executions.

Let 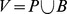. The state 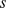 of IHM 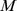 is a vector of length 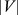, where entry 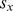 is the number of tokens in 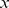, if 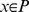, and the Boolean value of 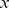, if 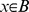. As the state of the system evolves as transitions, Boolean functions, and triplets in 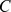 are executed, we denote by 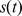 the state of 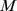 at time 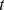. In particular, 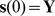. In other words, 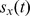 denotes the value of variable 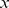 at time 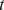.

Let 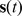 be the state of the system at time 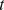. Petri net transitions and Boolean functions are executed at time 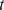 according the rules described above. The state of variable 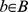 that is an element of a triplet 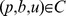 is updated as follows:
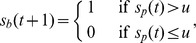
and the state of a Petri net place 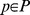 that is an element of a triplet 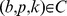 is updates as follows:
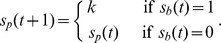
In other words, in the Petri-to-Boolean conversion, we set the Boolean variable to 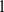 if the number of tokens in the place exceeds the given threshold 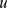, and to 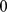 otherwise. For the Boolean-to-Petri conversion, we set the number of tokens in the place to value 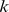 if the Boolean variable has state 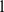, and keep the state unchanged otherwise. The choice of the values of 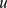 in the Petri-to-Boolean is not straightforward and must be learned from the data. We discuss below how we set the thresholds for our two specific biological systems.

#### Execution of the full IHM

As discussed above, once the biological system is modeled using the IHM, it is straightforward to execute it. However, as the three biological components operate at different time scales, the execution of the integrated hybrid model must account for this by introducing delays into the execution protocol. Here, we describe the additional details of execution, as they pertain to the full IHM model.

To execute the full IHM, we make use of a *global clock*, or simply clock, that governs the execution of transitions and Boolean functions, and a *priority queue*, or simply queue, for simulating delays that capture the differences in time scales. At each tick of the clock, each of the three components (Petri net, Boolean network, and triplets), updates its state, resulting in an update to the entire state of the system. The order in which the three components update their states is random and thus changes from one clock tick to the next. This is a rather simplistic approach to incorporating stochasticity and concurrency in the model; nonetheless, we show below that it works very well on the two biological systems we consider here. We now describe how each of the three components is updated in each tick of the clock, which is similar to the general description above, yet with some minor additional details.

The Petri net component is updated according to Gillespie's first reaction method. The only difference is that to obtain state 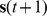 from state 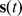, we average the execution of the Petri net component over 20 times. More formally, we execute Gillespie's algorithm 20 independent times, each starting from state 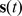, thus producing 20 candidates for 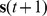. We then average these 20 candidates to produce a single next-state 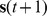, which is the state of the Petri net component at the end of the clock tick. This averaging approach was used before and shown to produce good results when simulating signaling networks using Petri nets [32]. The use of Petri net under a global clock is similar to the the timed Petri net model [42].

The Boolean network component is updated synchronously with necessary modifications to suit the use of a global clock. In every clock tick, each Boolean variable that is not on the queue and whose state changes from 0 to 1 at that clock tick is put on the queue with state 1 with a time delay 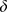 chosen uniformly in the range 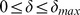. As the global clock ticks, the time delays of all items on the queue decrease, and whenever the time delay of a Boolean variable reaches 0 at a clock tick, the new state of the variable (which is 1) becomes visible to the system. More formally, let variable 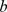 be added to the queue at time 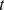 with delay 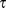. Then, the state of variable 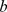 in its duration on the queue is given by
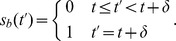
If a Petri-to-Boolean triplet is chosen to execute in a given tick of the clock, then it executes instantaneously, according to the rule described above. If a Boolean-to-Petri triplet is chosen to execute, it is executed with time delay, in a similar fashion to the Boolean network component. That is, the triplet is added to the queue with a time delay, and when the time delay expires, the triplet is evaluated and the value of the Boolean variable is updated.

Given the stochastic nature of the execution, the model must be executed multiple times and the results are averaged. While the syntax and semantics, as produced by the execution strategy, are general enough, the specifics of the model in terms of the connectivity and parameterization are determined by the biological system under consideration. Below, we use our new modeling approach on two biological systems. We describe for each of the two systems the connectivity and parameters that we used.

#### Putting it all together

The construction of an IHM for a biological system entails four steps:

1. First, the connectivity map of the network under consideration is assembled. This can be achieved by mining the literature for connections relevant to the network, or by making use of information from public databases.
2. Second, the network elements are mapped to the individual IHM components. As described above, signaling and metabolic elements are mapped to a Petri net component, transcriptional elements are mapped to a Boolean network component, and the relevant connections between the two are established using triplets.
3. Third, the resulting model is parameterized. This requires establishing the Boolean functions in the Boolean network component, establishing the thresholds in the connection triplets, and setting the rates for transitions and the values for the mapping 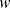 in the Petri net component for determining the number of tokens passed between places and transitions.
4. The start state 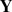 of the system is set. This is determined based on the experiment or question that is being investigated.

Once these four steps are carried out, the resulting IHM can be executed using the strategy described above and the dynamic trajectories can be obtained and analyzed.

## Results

In this section, we demonstrate the application of our new modeling approach on two biological systems: transcriptional regulation of glucose and Osmoregulation in *S. cerevisiae*. Both biological systems intrinsically involve metabolic, signaling, transcriptional regulatory components and complicated interaction in-between these components. They cannot be comprehensively modeled using traditional frameworks that specifically targets separate cellular components.

### Transcriptional regulation of glucose

In order to assess the ability of IHM to capture the dynamics of complex biological systems we implement an IHM model for the system of transcriptional regulation of glucose metabolism, which was surveyed in [1]. Timely uptake of cellular glucose from the blood, a task regulated by the secretion of insulin and glucagon, is crucial to human metabolism. This system involves the interaction of multiple cellular components in cells of different cell types and cells that span a physical distance. We demonstrate that IHM can readily be adapted to model such a biological scenario, and allows us to investigate issues such as the interplay between AKT and FOXO in this system. Given that the modeled system involves more than a single cell type, it is unclear how to apply FBA-based techniques to it.

#### Assembling the connectivity map

**Figure 2:**
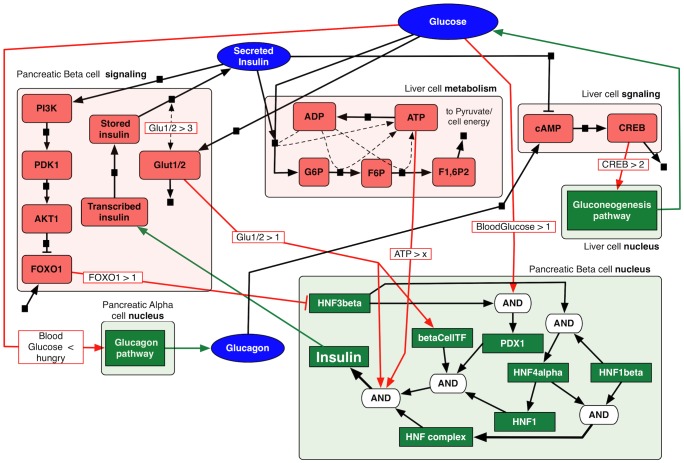
Graphical representation of glucose system. Red shapes are Petri net places (signaling and metabolism), and small black squares on the arrows represent Petri net transitions (dashed lines correspond to enzymatic interactions). Green squares are Boolean network elements for regulatory components. Blue ovals are also Petri net places and correspond to interconnection elements. The Petri-to-Boolean arithmetic conditions are noted on/through red arrows (specific values are defined in the section of parametrizing the model). The Boolean-to-Petri connections are indicated with green arrows. The initial condition defined by vector 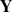, is set as follows: all Petri net places have 0 tokens except *ADP* (10 tokens) and *Glucose* (20 tokens); all Boolean network elements are set to 0, except *HNF3beta* and *HNF1beta*, which are set to 1. The ‘
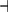
’ connections into Boolean variables correspond to the negation functions. For the Petri net component, the ‘
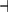
’ connection from transition 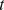 to place 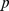 is a schematic representation of inhibition, which is implemented using the standard Petri net definition as 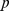 being an input place to transition 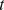. Transitions without inputs or outputs represent sources and sinks, respectively.

Desvergne *et al.* reviewed the transcriptional regulation of the insulin gene under different levels of blood glucose. At a high blood glucose level (for example after feeding), the insulin gene is transcribed in pancreatic 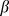-cells and released to tissues, such as liver and muscle, to uptake glucose from the blood. When blood glucose is low, glucagon is secreted from pancreatic 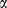-cells to bind the liver cell receptor to decompose stored glycogen into glucose, maintaining the blood glucose level as is necessary for tissues such as the brain. The response of glucagon signal by the liver cell is effectuated by the signaling pathway of extracellular signal-regulated kinase (ERK) [43], cAMP-dependent protein kinase (PKA) and cAMP-response element-binding protein (CREB) [44], [45]. The mechanism by which glucose and insulin, independently or together, modulate insulin gene transcription involves the sensing of the blood glucose level by both the pancreatic 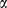 and 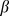-cells in their transcriptional network. The transcriptional regulation of insulin is mediated by a handful of transcriptional factors including FOXO [46], [47], PDX1 [48], [49] and hepatocyte nuclear factors (HNFs) [50]–[52]. Blood insulin also triggers the PI3K-AKT1 pathway [53] which inhibits FOXO and promotes the insulin production in tissues including liver and pancreatic 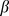-cell [54]. By collecting from the aforementioned literature all the necessary information about interactions within and between different components, we manually constructed the connectivity map of the network, which is the first step toward constructing the integrated hybrid model. Figure 2 shows the connectivity map, which includes intracellular interactions between the liver, pancreatic-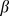, and pancreatic-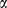 cells.

#### Parameterizing the model

Once the topology was acquired, a minimal amount of custom parameterization is needed. Most of the parameters were set in a very simple way, with some exceptions that we describe in detail below:

- Rates of all Petri net transitions are set to 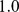 for all internal transitions and to 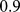 for all sink and source transitions.
- The maximum number of tokens that a place can have is 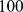.
- The values of 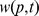 and 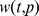 were set to 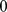 for every pair 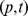 or 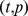 that does not have a connection in Figure 2. For the rest of the place/transition pairs, we set the 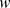 value to 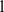.
- For the Boolean network and connection triplets, for Petri-to-Boolean connection, the thresholds are shown in Figure 2; for Boolean-to-Petri connections, we used 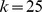.
- For the time delay, we set 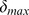 to 20

In addition to these general rules, the following parameters were fine-tuned to simulate the conditions of the feed and fast cycle used for validating the model (described in the following section).

1. In the Petri net component that corresponds to liver metabolism, the values of 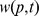 and 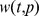 for every pair 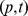 or 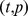 that *does* have a connection in Figure 2, we set the 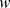 value to 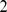.
2. In the pancreatic 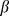 cell's signaling, the Petri net connection involving transition 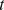 between *Blood glucose* and *Glut1/2* sensor has the weights 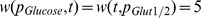.
3. In the pancreatic 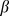 cell's signaling, the Petri net connection involving transition 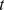 between *Stored Insulin* and *Secreted Insulin* requires an input of the read-arc from *Glut1/2* with weights 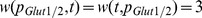.
4. The arithmetic condition between *ATP* and the pancreatic 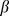 cell's nucleus is evaluated based on 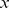 tokens, where 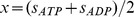, which is a constant value due to the conservation of tokens between these two places.
5. A mechanism to facilitate feed/fast cycle is not considered to be a part of the IHM static description, but is needed to sustain feed/fast cycle, by introducing the following two invariants, which are tested at every time step.
  - *Feeding* is activated when the system is determined to be in the *starvation* state. At each time step the condition (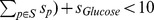, where 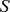 is a set of places for liver metabolism, is tested. If the condition evaluates to *true*, 2 tokens are deposited into the *Glucose* place for the next 15 steps; otherwise, no action is taken.
  - *Fasting* is activated when the system is determined to be in the *hungry* state. In this state, the pancreatic-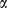 cell is activated in response to low *Glucose* (below the *hungry* threshold of 20 tokens). In this state, the Boolean-to-Petri connection associated with *Glucagon* and *Transcribed insulin* are adjust by continuously reducing 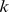 by a small amount (uniform random value 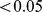). In the *feeding* state, the original 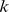 values associated with these connections are restored.

#### Model validation and results

To validate the performance of our model, we focused on modeling the regulation of insulin and glucose production by means of the interactions between and within the liver cell and the pancreatic 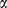, and 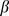 cells under the conditions of *high* and *low* glucose. Both of these scenarios can be captured by modeling the oscillatory behavior of the feed and fast cycle—the phenomenon frequently used in analyzing glucose and insulin regulation.

To simulate the feed and fast cycle, we differentiate between two conditions: *hunger* and *starvation*. The former condition is defined by the low blood glucose and triggers the release of glucose by the liver via gluconeogenesis, whereas the latter condition is characterized by much lower glucose and promotes manual “feeding.” In the phase of fasting, the activity of liver glucose production slows down proportionally to the time duration since the last manual feeding, allowing the system to transition into the *starvation* condition as the liver becomes less effective.

**Figure 3:**
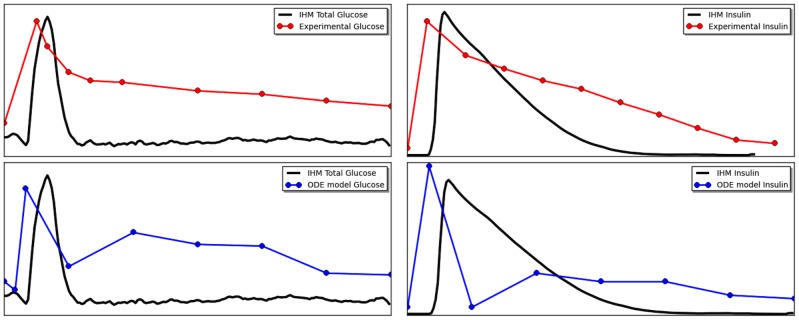
Comparison of blood glucose (left) and insulin (right) dynamics in a single fast/feed cycle as simulated by our model with the experiential data by Korach-André *et al* [**55**] (top) and ODE-based model by Liu *et al* [**56**] (bottom). Our IHM is shown in solid black line. Experimental data and ODE model results are reconstructed from Figure 7 in [56]. The results of IHM dynamics qualitatively match to both experimental data and ODE-generated data.

We validate our model against feed/fast cycle from two sources, as shown in Figure 3. The experimental data of glucose circulation by Korach-André *et al* [55] (red lines on the top two plots) and an ODE-based model of the simplified regulator system of blood glucose by Liu *et al* [56] (blue lines on the bottom two plots) are compared to our IHM outcomes (solid black line in all plots). The IHM results are averages of 100 stochastic executions of the entire system, as described above. The plots show good match between the dynamics of IHM and both experimental data and ODE model. In fact, the slower insulin absorption in IHM is closer to experimental data than the results of the ODE model. This validation shows IHM's efficacy in capturing the dynamics of glucose absorption and insulin secretion even in light of complex model dynamics. The concentrations on the x-axis are measured as the number of tokens, and time is measured in arbitrary units.

**Figure 4:**
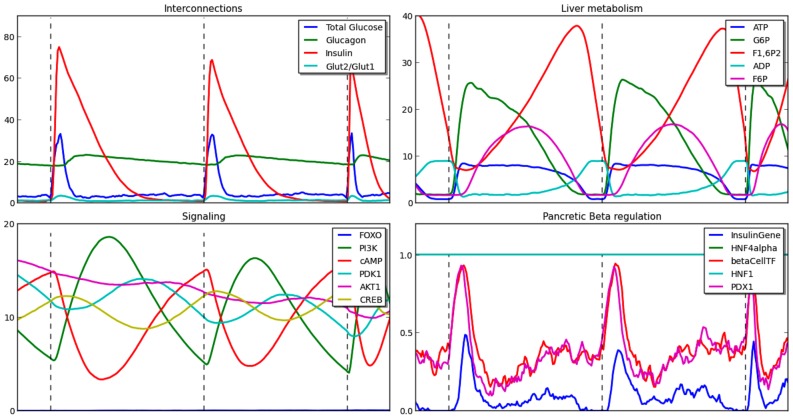
The dynamics of all components in the IHM for feed/fast cycle in for the transcriptional regulation of glucose metabolism. The plots show selected species from different components — component interconnections (top left), selected species from liver metabolism (top right), selected species from pancreatic beta-cell and liver signaling (bottom left), and selected species pancreatic beta-cell regulation (bottom right). X-axis for Petri net components are expressed in tokens, and for Boolean component is an average of Boolean values, 0 and 1.

Further, we observe the complete dynamics of this system. Figure 4 shows the dynamics of all the components of the model and their interconnections. The dashed lines indicate the beginning of manual feeding (the start and end of a single feed/fast cycle). The model is an average of 100 individual simulation runs. We can see from the interconnection plots that insulin spikes when glucose is added in the system during feeding. The insulin is slowly utilized as the system switches into fasting phase and maintains the lower level of glucose through the liver. Additionally, the level of glucagon increases as soon as the feeding ends and levels of glucose start to drop, resulting in hepatic glucose production and maintaining of glucose inside the normal range [57]. The liver metabolism plot shows a clear propagation of glucose through the system. As the fluxes through the system becomes too low, it contributes to the feeding condition and the next feed/fast cycle. In the dynamics of pancreatic-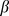 cell regulation, we can clearly see a stronger response form insulin gene expression during the feeding, and more moderate insulin production in response to glucose produced by the liver during fasting. Lastly, signaling plot (which contains species form liver and pancreatic-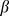 cell on one plot) shows the the activation through PI3K
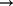
AKT1
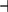
FOXO pathway, which is known to be a positive feedback from insulin to promote further regulation of insulin production. Particular, the activity of this pathway prevents FOXO1 from exhibiting negative feedback onto the 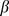-cell regulation, which, in turn, would impair the production of insulin.

It has been a long standing hypothesis that glucose is a regulator of FOXO1 through the insulin receptor. FOXO1 is found to be a central player in 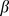-cell compensation of insulin resistance [58]. FOXO1 negatively regulates insulin expression in 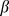-pancreatic cell [47]. In type-2 diabetic population, the insulin production does not meet the metabolic demand [59]. The positive feedback, comprised of insulin
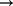
PI3K
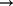
AKT1
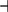
FOXO
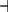
PDX1
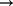
insulin, helps 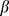-cell mitigate the difference between the demand and supply of insulin at the initial stage of diabetes. A sustained execution of this pathway leads to failure of 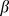-cell as is characterized by the reduction of mass and number of 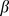-cells [60]. This failure is closely related to PDX1 [61]. Bernel-Mizrachi *et al.* provide time series data, comparing the feed and fast cycle in the normal condition and under reduced Akt, which is responsible for suppressing FOXO1 [62]. If the concentration of Akt1 is low, FOXO1 becomes active and the insulin expression is expected to be lower than normal, and, therefore, the absorption of glucose slows down. We are interested in testing this hypothesis using our integrated hybrid model for this system.

**Figure 5:**
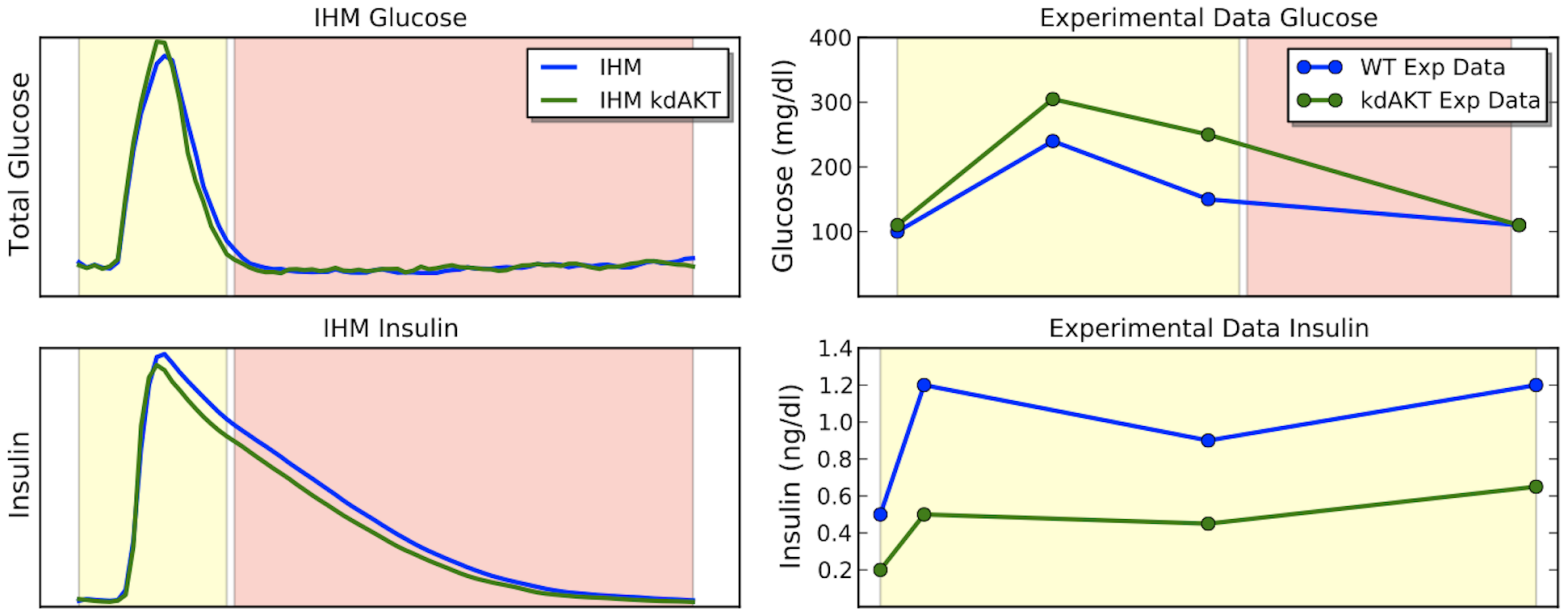
The dynamics of IHM under normal Akt and reduced Akt (kdAkt) as compared to the experimental data in [**62**] (**Figures 2B and 2D** in [**62**]). The kdAkt experiment was modeled by IHM by reducing the rate at which Akt suppresses FOXO and increasing the rate of the source transition into FOXO. In all images, yellow background indicates feeding stage, and red corresponds to fasting. The experimental data measures the glucose levels at the feeding stage and insulin secretory response during fasting. IHM shows the entire cycle. We observe the glucose of kdAkt model being higher than normal condition, as well as lower insulin secretion in reduced Akt scenario. These results correspond to the observations in the experimental data.

The reduction of AKT activity on FOXO was achieved by reducing the rate of AKT1
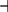
FOXO reaction, while also increasing its source reaction rate (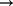 FOXO). The dynamics of IHM under normal Akt and reduced Akt (kdAkt) are compared to the experimental data from [62] in Figure 5. In all images, yellow background indicates feeding stage, and red corresponds to fasting. The experimental data measures the glucose levels at the feeding stage and insulin secretory response during fasting. IHM shows the entire cycle. We observe the glucose of kdAkt model being higher than normal condition, as well as lower insulin secretion in reduced Akt scenario. These results correspond to the hypothesis and the observations in the experimental data.

**Figure 6:**
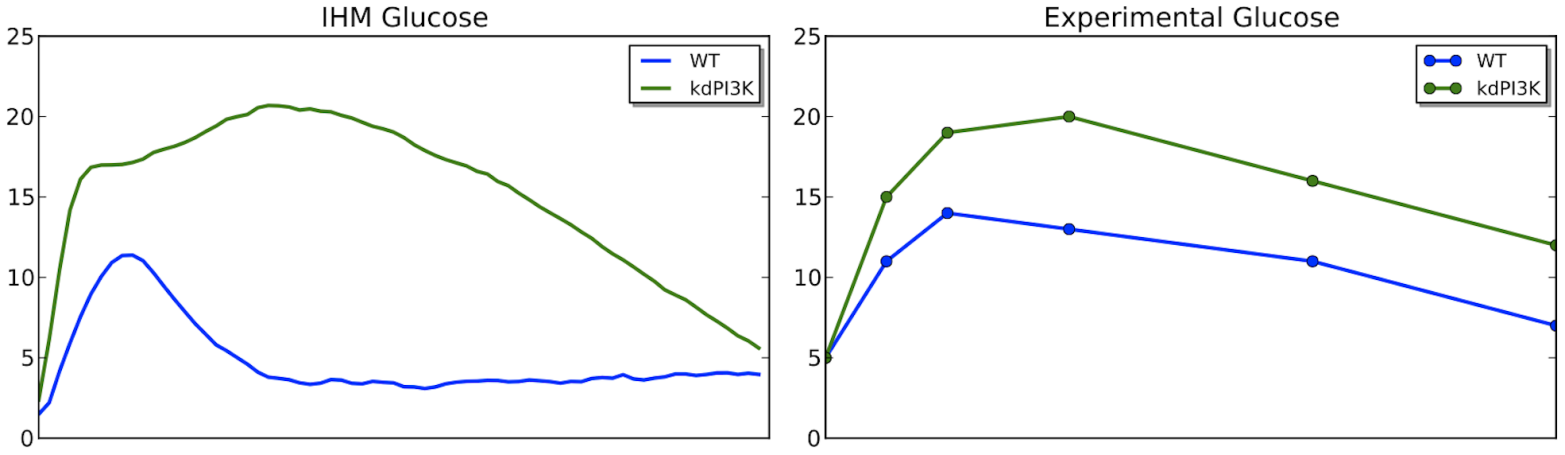
Glucose response from PI3K inhibition. The comparison between IHM model (left) and experiment (right). Inhibiting PI3K was modeled by setting rate between *Secreted insulin* and *PI3K* to 0. In IHM model, glucose is higher with PI3K inhibition which is consistent with experimental data. The experimental data is reconstructed from Figure 3C in [63].

PI3K kinase has been shown to be able to induce the insulin secretion (PI3K
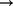
AKT1
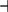
FOXO
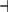
PDX1
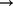
insulin) which further facilitates glucose uptake. Inhibition of PI3K results in the accumulation of blood glucose. In order to validate our model against this scenario we inhibit PI3K kinase in our IHM model of the glucose metabolism system and compare the result to experimental data extracted from [63]. As is shown in Figure 6, IHM correctly recovers the accumulation of blood glucose. The dynamics resembles the experimental data. In the experiment, mice were first treated with pan-PI3K/mTOR inhibitors PI-103 [63] before glucose is administered. We set the initial concentration of PI3K to zero to simulate this treatment.

### Osmoregulation in *S. cerevisiae*

Yeast responds to the environmental osmolarity by adjusting the cellular glycerol concentration [37]. Such response is mediated through signaling pathways that sense the extracellular osmotic pressure as well as transcriptional regulation of about 10% of the yeast genes that manipulate the metabolism of glycerol. The effect of the medium osmolarity is first sensed and transmitted by the well-studied HOG/MAPK pathway [64], [65] whose upstream involves two redundant branches—Sho1 branch [66], [67] and Sln1 branch [68]. The HOG signaling pathway is one of the first to sense the osmotic upshift, playing a pivotal role in yeast's adaptation to high osmolarity. Hog1, the end effector of HOG pathway, activates in the nucleus the central transcriptional factors Hot1 [69], Msn2/4 [70] and Ptp2/3 [71]. These transcriptional factors turn on the expression of enzymes that promote glycolysis, which leads to the production of glycerol, an inert osmolyte. The surge in the glycerol concentration increases the cytosolic osmolarity, counteracting the osmotic upshift in the environment and protecting the cell from dehydration. While the effect of Gpd1/Gpp2 (which is a product of Hot1 and MSN2/4) gene controls osmoregulation via glycerol production though metabolic pathway, Ptp2/3 is a much stronger mediator of osmotic stress, as it acts on suppressing the activity of Hog1 transcription factor directly.

#### Assembling the connectivity map

**Figure 7:**
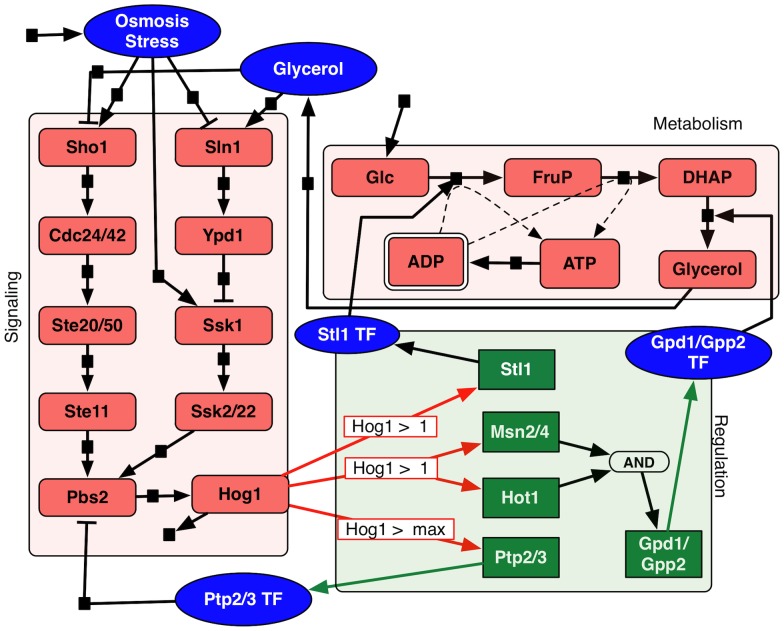
Diagram of the *S. cerevisiae* HOG pathway. Graphical representation of glucose system. Red shapes are Petri net places (signaling and metabolism), and small black squares on the arrows represent Petri net transitions (dashed lines correspond to enzymatic interactions). Green squares are Boolean network elements for regulatory components. Blue ovals are also Petri net places and correspond to interconnection elements. The Petri-to-Boolean arithmetic conditions are noted on/through red arrows (specific values are defined in the section of parametrizing the model). The Boolean-to-Petri connections are indicated with green arrows. The initial condition defined by vector 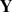, is set as follows: all Petri net places have 0 tokens except *ADP*, which has 10 tokens; all Boolean network elements are set to 0. See caption of Figure 2 for more details about the representation.

Like in the previous case, we first constructed an integrated hybrid model for the system of *S. cerevisiae* HOG pathway by manually collecting information from a previous curation by Lee *et al* [10] and additional literatures referred above. Figure 7 provides a visualization of this model. Unlike the previous example, this model focuses on the interplay within a single cell type. In addition to the clear interaction between signaling and transcriptional regulation via Hog1 and the provision of enzymes from the transcriptional regulation to the metabolic component, we also see a feedback of glycerol to the signaling component, a communication between signaling and metabolic components via metabolites.

#### Parameterizing the model

Most of the parameters in this IHM are set to the exact same default values that we used in the previous example, with only two instances, where the specific behavior needed to be fine-tuned.

1. In signaling the Petri net connection 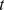 between *Ptp2/3 TF* and *Pbs2* has different weights 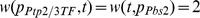.
2. For the Boolean network and connection triplets, for Petri-to-Boolean connection, the thresholds are shown in Figure 7; for Boolean-to-Petri connections, we used 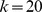.
3. The source transition for *Osmosis stress* is set to 100 when simulating “stress” condition and to 0 when simulating “no stress” condition.

#### Model validation and results

**Figure 8:**
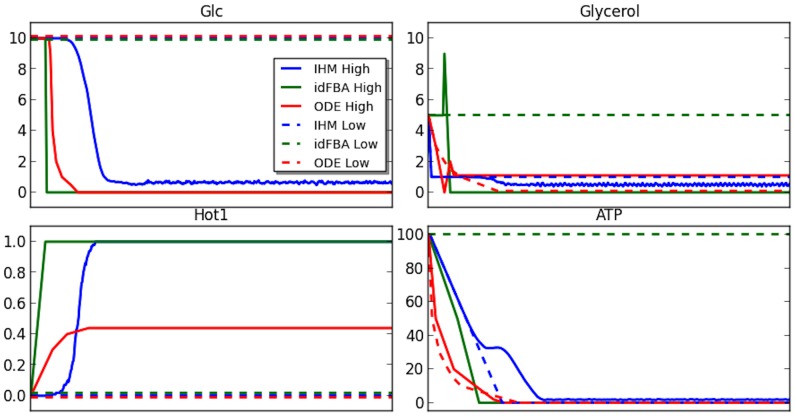
Validation of our model against idFBA and ODE-based model as generated by Lee *et al* [**10**] (contrast to **Figure 9** in [**10**]). The plots show the dynamics under osmotic stress (solid lines), and under no osmotic stress (dashed lines). The colors on all plots are indicated in the top left panel. The correspondence in qualitative behavior for all solid lines indicate similar results for all models under osmotic stress; for all dashed lines indicate similar results for all models under no osmotic stress.

This particular system was analyzed by Lee *et al* with the idFBA model [10]. Using this example, we compare the performance of IHM and idFBA. Figure 8 shows the behavior of the models under high and low osmotic stress conditions. The behavior of the selected species in the system corresponds to those validated by Lee *et al* [10]. The results of three models is depicted: IHM, idFBA model, and kinetic based model for this system. The plots show the behavior of the system in the presence of high osmotic stress (solid lines) and low osmotic stress (dashed lines). The results of IHM corresponds well with idFBA in all of these species in the high osmotic stress case. However, there are two discrepancies in the low osmotic stress case. In our model, both ATP and *Glycerol* go down, while in idFBA they stay constant. The former decline can be easily explained by the transition in IHM where all of the ATP gets converted into ADP in the presence of no metabolic activity. The latter decline is explained by the topology, where any available *Glycerol* (the initial concentrations in this case) will always be used for activating *Hog1*. Overall, our integrated model achieves comparable results by just mainly relaying on the topology of the system. In addition, while IHM is a stochastic model and requires averaging, a single result take just a few seconds with an average of 100 iteration taking no longer then 5 minutes. In idFBA, on the other hand, many parameters are involved in executing ODEs.

**Figure 9:**
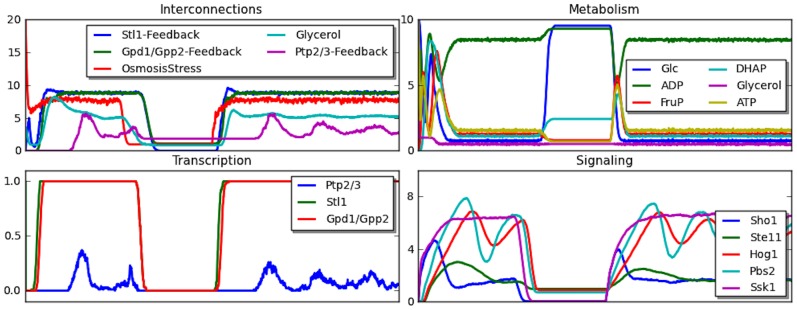
Dynamics of IHM for all components of HOG pathway under osmotic stress. The plots show selected species from different components — component interconnections (top left), species from metabolism (top right), selected species from regulation (bottom left), and selected species signaling (bottom right). X-axis for Petri net components are expressed in tokens, and for Boolean component is an average of Boolean values, 0 and 1.

We further analyzed the complete dynamics. Figure 9 show the dynamics of all the components in the HOG pathway in the cycle of presence of osmotic stress, followed by the absence of osmotic stress, and finally under the osmotic stress again. We can clearly see the activity of Hog1 pathway in the presence of osmotic stress, and its idleness under no stress condition. During the osmotic shock, Gpd1/Gpp2 feedback is always on as it mediates the production of glycerol. However, it can be seen from the plot, that when the concentration of Hog1 becomes too high, it turns on Ptp2/3 feedback that directly suppresses signaling of Pbs2, slowing down the response to osmotic stress. Also, the Stl1 stays expressed during osmotic stress and regulates the activity of metabolism.

## Discussion

We proposed a simple, yet effective, integrated hybrid model (IHM) that allows for simultaneously modeling *signaling*, *metabolic*, and *regulatory* processes within a single framework, while explicitly capturing the dynamics within each component and the interplay among them. As we applied the integrated model to two biological systems, we demonstrated how much our model can capture by mainly relying on the topology of the system (given the simple and general rules for setting most of the model's parameters). In both systems, we were able to successfully validate our results against both experimental data and other models. In the case of transcriptional regulation of glucose, we compared our model against an ODE-based model that only focuses on glucose-insulin interactions, while in our case we consider a larger system. The results compare well against the experimental data. No comparison was done with other integrated models, since it is not clear how to formulate an FBA-based model for this system. In the case of the osmoregulation system, we compared our model against the idFBA approach [10].

The IHM framework has an intuitive graphical representation that makes the construction of the connectivity map of the model a relatively simple task. Further, as experimental evidence becomes available to provide support for new connections or against existing ones, the connectivity map can be readily updated to accommodate this new evidence without having to recreate the model from scratch. Our model is reconstructible and its parameterization is obtainable from qualitative data, which is abundant in the literature and public databases. It is important to note that while the connectivity map is often easy to obtain from the literature and public databases, parameterizing the IHM poses the biggest challenge in terms of obtaining the executable model. In this paper, we parameterized the IHM for both biological systems manually—a task that took very short time to achieve, given that most of the parameters were set using general rules and only a few of them had to be fine-tuned. The results (e.g., the feed/fast cycle in the regulation of glucose metabolism system) are qualitatively robust to most parameter values that we choose, as tested by executing the model with parameters varied around the chosen value. We identify as a direction for future research the task of devising computational techniques for automated parameterization of our IHM using qualitative experimental data. Some techniques for a similar task were recently introduced [72] and we will build on those.

While the aforementioned existing approaches for integrated analysis of biological networks provide promising frameworks, a salient feature of all of them is that they depend on flux-balance analysis (FBA) as a main analytical component. This dependence means that an FBA model must be curated for the system under analysis, which is not clear how to obtain for a system such as the regulation of glucose metabolism, which involves more than a single cell type. Further, this dependence necessarily makes the analysis metabolism-centric and shifts the focus from the other two components. Third, as FBA is aimed at understanding the behavior of the system at *steady state*, the dynamics of the system cannot be studied, except under the idFBA modeling technique, as it takes a step-wise approach to conducting FBA. Our model, on the other hand, is not based on FBA and, consequently, provides a complementary approach to the FBA-based ones.

Our model builds on the success of Boolean networks and Petri nets for modeling cellular networks. As advances continue to be made for both modeling techniques, our integrated modeling approach would readily benefit from these advances, as different flavors of of Boolean networks (e.g., probabilistic ones) and Petri nets (e.g., colored Petri nets) can be plugged into our model without having to modify the way the connectivity map is constructed or the system is executed. In other words, our model can be viewed as a reconfigurable model, where different components, along with their execution protocols, can be assembled to generate a model of integrated systems.

It is important to note that while we made decisions on the model to fit the two biological systems we studied, other biological systems may require more features in the modeling approach. For example, in the Petri-to-Boolean connections, it might be the case that the state of the Boolean variable is set based on a *function* of a set of the Petri net places. Our IHM can be easily extended to incorporate such features, with little or no need to modify the execution strategy. That is, the model is easy to extend as long as the syntax of the new features and their effects on the execution strategy are well-defined.

Last but foremost, our IHM approach lends itself in a straightforward manner to hypothesis generation. Perturbation experiments can be simulated *in silico* by setting the numbers of tokens at Petri net places and Boolean variables to a certain value, and the system can be executed to study the effect. For example, a Boolean variable can be set to 0 to simulate its inhibition, or the number of tokens can be set to a large number in place to represent a constitutive enzyme. Further, new components can be added in or existing ones can be removed easily to study the effect of these components on the overall performance of the system. Finally, while we chose to model transcriptional regulation using Boolean networks here, the entire system (that is, all three types of biological networks) could be represented using a single Petri net. This allows for a more refined simulation of the transcription factors and their targeted genes, but also requires replacing the Boolean functions by Petri net transitions whose parameters must be learned from the data.
